# Cell Wall Surface Properties of *Kluyveromyces marxianus* Strains From Dairy-Products

**DOI:** 10.3389/fmicb.2019.00079

**Published:** 2019-01-31

**Authors:** Giorgia Perpetuini, Fabrizia Tittarelli, Giovanna Suzzi, Rosanna Tofalo

**Affiliations:** Faculty of Bioscience and Technology for Food, Agriculture and Environment, University of Teramo, Teramo, Italy

**Keywords:** *Kluyveromyces marxianus*, *FLO11*, *STE12*, adhesion properties, biofilm formation, mat structure, RT-qPCR

## Abstract

Thirty-three *Kluyveromyces marxianus* strains were tested for the ability to form biofilm and mat structures in YPD and whey and for cell surface hydrophobicity. To identify genes potentially involved in adhesion properties, a RT-qPCR analysis was performed. Eight strains were able to adhere on polystyrene plates in both media and formed a mature mat structure. These strains showed a different level of hydrophobicity ranging from 55 to 66% in YPD and from 69 to 81% in whey. Four *K. marxianus* orthologs genes (*FLO11*, *STE12*, *TPK3*, and *WSC4*), known from studies in other yeast to be involved in biofilm formation, have been studied. *FLO11* and *STE12* showed the highest fold changes in all conditions tested and especially in whey: 15.05 and 11.21, respectively. *TPK3* was upregulated only in a strain, and *WSC4* in 3 strains. In YPD, fold changes were lower than in whey with *STE12* and *FLO11* genes showing the highest fold changes. In mat structures *FLO11* and *STE12* fold changes ranged from 3.6–1.3 to 2–1.17, respectively. Further studies are necessary to better understand the role of these genes in *K. marxianus* adhesion ability.

## Introduction

Many microorganisms can form multicellular, sessile, surface-bound assemblies known as biofilms. Microorganisms able to form biofilm can shift between two different forms: planktonic cells in a free-living state, and sessile cells in a multicellular state (Andersen et al., 2014). The ability to form biofilm is strictly related to another multicellular development, the so-called mat structures. Mat is a structure composed of a complex of aggregated cells. It is formed of a network of multiple radial spokes originating from a central hub. This elaborate pattern is regulated by specific processes, depending on specific transcriptional programming, environmental signals, and cell–cell communication systems (Reynolds, 2006). Most of the studies regarding yeast adhesion were principally focused on *Candida albicans* (Chaffin et al., 1998; Hostetter, 1999; Gaur and Klotz, 2004; Otoo et al., 2008; Granger, 2018) and *Saccharomyces cerevisiae* (Bowen et al., 2001; Reynolds and Fink, 2001; Pérez-Torrado and Querol, 2016; Di Gianvito et al., 2017, 2018). Adhesion properties have mostly been described by the action of cell wall glicoproteins (Verstrepen and Klis, 2006; Goossens and Willaert, 2010; Moreno-García et al., 2018).

However, recently there is an increasing interest for some non-*Saccharomyces* species which can be exploited in foods fermentation for their technological traits. Among them of interest is *Kluyveromyces marxianus* (Fonseca et al., 2008; Lane and Morrissey, 2010). Each *K. marxianus* strain has diverse properties and characteristics, in fact it is not possible to establish an exclusive *K. marxianus* strain characterized as a “model” (Lane and Morrissey, 2010; Tittarelli et al., 2018). It has been reported that *K. marxianus* was the principal species in Pecorino di Farindola and Parmigiano Reggiano cheeses, showing genotypic and phenotypic polymorphisms (growth kinetics in whey, production and degradation of organic acid, amino acid consumption, production of aromatic metabolites) (Tofalo et al., 2014; Fasoli et al., 2015; Coloretti et al., 2017; Tittarelli et al., 2018). Moreover, a recent study showed differences among *K. marxianus* isolates from a fermented goat milk of the Yaghnob Valley based on mat formation and adhesion properties (Perpetuini et al., 2018a). Despite the efforts done, genetic studies combined with phenotypic analyses are necessary to fully exploit *K. marxianus* strains and highlight some understudied features such as the genetic basis underlying adhesion properties. However, biofilm formation by microorganisms is a multispecies feature, and lactic acid bacteria (LAB) are able to colonize surfaces by forming biofilms as well (Piard and Briandet, 2015; Arena et al., 2017). Although the biofilms formed on food by LAB usually spoil the products and damage both equipment and working surfaces (Flemming and Wingender, 2010), in some manufactures (like cheese manufacture) biofilms are advantageous for the food technology, being also essential for achieving the uniqueness of the product (Arena et al., 2017). The presence of yeasts and bacteria in co-culture may affect the individual biofilm production. However, the molecular mechanisms involved in adhesion of *K. marxianus* are almost unknown.

The aim of the present study was to explore the capacity of 33 *K. marxianus* strains isolated from different geographic and technological origins, to form biofilm and mat structures in different conditions (YPD and whey) and determine the hydrophobicity of cell surface. Moreover, a RT-qPCR analysis was performed to identify genes upregulated in sessile cells.

Particularly, we focused on genes that are already known in *S. cerevisiae* to be involved in biofilm formation: *FLO11* gene – in *Kluyveromyces lactis* it showed some similarities with *S. cerevisiae* YIR019C *MUC1* GPI-anchored cell surface glycoprotein essential for pseudohyphal formation and invasive growth (Van Mulders et al., 2009; Legras et al., 2016); *STE12* – the transcription factor activated by a MAP kinase signaling cascade and the activity of the gene is involved in mating or pseudohyphal/invasive growth pathways; *TPK3* – involved in nutrient control of cell growth and division; *WSC4* – involved in cell wall integrity and stress response. Several interacting regulatory pathways control the adhesion transcriptional factors. The genes involved in these pathways are triggered by various environmental signals, such as carbon and nitrogen starvation, pH or stress factors (Verstrepen et al., 2003; Sampermans et al., 2005). Microorganisms are also able to change from non-adherence to adherence state as a mechanism of adaptation to stress factors (Verstrepen and Klis, 2006).

## Materials and Methods

### Samples Origin

Thirty-three *K. marxianus* strains isolated from different dairy products were considered in this study (Fasoli et al., 2016; Tittarelli et al., 2018). The strains are listed in Table 1. The strains were grown in YPD medium (yeast extract 1% *w*/*v*, peptone 2% *w*/*v*, glucose 2% *w*/*v*) overnight at 28°C.

**Table 1:** List of strains analyzed in the study.

Strains	Isolation source	Culture collection
LM15, LM17, LM20, LM28, LM38, LM42, LM44, LM72, LM114, LM116, LM127, LM133, LM136, LM142, LM148, LM153, LM161, LM167, LM169	Parmigiano Reggiano natural whey starter culture	Department of Agricultural and Food Sciences, (University of Bologna)
6M2, 1SC4, K326	Parmigiano Reggiano cheese	
M38, M48, M68, M81, M83, M135	Pecorino di Farindola cheese	Faculty of Bioscience and Technology for Food, Agriculture, and Environment, (University of Teramo)
VG1, VG4, VG6	Cow milk whey	
FM09	Fermented milk	
CBS 834^T^		CBS-KNAW, Westerdijk Fungal Biodiversity Institute (Utrecht, the Netherlands)
DMKU 3-1042, NBRC 1777, CCT 7735, DMB1, KCTC 17555, IIPE453, BO339	Genome sequences	A.N.: AP012220.1 A.N.: AP014606.1 A.N.: CP009310.1 A.N.: BBIL00000000.1 A.N.: AKFM00000000.2 A.N.: LDJA00000000.1 A.N.: LXZY00000000.1


All isolates were kept frozen at -80°C in the same growth medium supplemented with glycerol as cryoprotectant (20% *v*/*v*). The strains belong to the Culture Collections of the Faculty of Bioscience and Technology for Food, Agriculture, and Environment (University of Teramo, Italy) and of the Department of Agricultural and Food Sciences (University of Bologna, Italy).

### Adhesion Properties Determination

#### Biofilm Assay on Polystyrene and Cell Surface Hydrophobicity Assay

Yeast adhesion to polystyrene was evaluated in YPD (pH 6.5 ± 0.2) and pasteurized whey (4.8% lactose, 1.2% proteins and 0.75% fats; pH 6 ± 0.2) at 100°C for 30 min. Yeast strains were grown overnight at 28°C in YPD under aerobic conditions and harvested at an optical density (OD_600__nm_) of 1. A 96-well polystyrene plate was prepared with 200 μl of each media, and each well was inoculated with 10 μl of cell suspension. The plate was incubated at 28°C for 2 days. Non-inoculated wells were used as negative control. Cells attached at the bottom of wells were considered as sessile cells, while unattached ones, as planktonic. Biofilm formation was evaluated daily. After 2 days, attached cells were carefully washed with saline buffer solution and recovered (Perpetuini et al., 2018b). Both sessile and planktonic cells were serially diluted and plated on YPD for cell plate count. The analyses were performed in triplicate.

The microbial adhesion to solvent (MATS) test was performed to evaluate the cell surface hydrophobicity (CSH) of cells grown in YPD and whey, according to Bellon-Fontaine et al. (1996) using n-hexadecane.

#### Mat Formation

The capacity of the yeast strains to grow in multicellular or mat structures, was evaluated as described by Reynolds and Fink (2001). Strains were inoculated onto YPD soft agar plates (0.3% agar) with a toothpick and incubated at 25°C for 5 days. Plates with 2% (*w*/*v*) agar were used as negative controls. Three biological replicates were made.

#### Phylogenetic Analysis

To find the genes involved in biofilm production in *K. marxianus* species and to obtain the sequences, the Yeast Gene Order Browser (YGOB)^1^ (Byrne and Wolfe, 2005, 2006), *Saccharomyces* genome database (SGD)^2^ or BLAST on NCBI^3^ were used. The nucleotide and amino acidic sequences were aligned with a Muscle alignment, using the software Mega (Kumar et al., 2016), and displayed phylogenetically using a Neighbor-Joining method bootstrapped to 1000. The analyzed sequences belonged to the following species: *Saccharomyces kudriavzevii*, *Saccharomyces uvarum*, *S. cerevisiae*, *Saccharomyces mikatae*, *Naumovia castelli*, *K. lactis*, *K. marxianus*, *Lachancea waltii*, *Lachancea thermotolerans* and *Lachancea kluyveri*.

The genes in the dataset were represented by the KEGG pathways. A search for each gene, specific for *K. marxianus* was done online in the KEGG pathway database^4^.

#### Genes Expression and RT-qPCR

Appropriate specific primers were designed using the online program Primer 3 Plus^5^. Specificity and properties of each primer set were checked with a BLAST search (Basic Alignment Search Tool^6^) and with Oligo analyzer 3.1 software. The primer sequences, amplicon size and qPCR program are shown in Table 2.

**Table 2:** Primers and RT-qPCR conditions used in this study.

Primer	Primer sequence (5′–3′)	Amplicon size (bp)	PCR condition
FLO11 F	TGGGTGCAGGACAACATTTA	171
FLO11 R	GTCGGTTGGGTTGTCAATCT		
STE12 F	AGATGAGGTGCCAGCTGAGT	215	
STE12 R	TCATTCCGTCCATGTCTTCA		
TPK3 F	TGGTTCAGGCAGTGTCTACG	152	5 min at 95°C, 40 cycles at 95°C for 30 s, 59°C for 45 s, 72°C for 45 s
TPK3 R	CGTGTCACCAATGTTGGAAG				
WSC4 F	GTGAGCTCGTGCAGTCACAT	206	
WSC4 R	CCGGAACTGGATAATGTCGT		
ACT1 F	GGCTGAACGTGGTTACTCCT	115	
ACT1 R	AGAAGCGGTTTGCATTTCTT		
ALG9 F	CCATCTCAGGATCCCTCTTC	147	
ALG9 R	GCATTCCAGCGAATAGTTGA		


Specificity of the PCR reactions was analyzed by melting curves temperature analysis. To evaluate genes expression RNA was extracted using the Trizol reagent method (Sigma-Aldrich, Saint Louis, MO, United States), following the manufacturer’s instructions.

The absence of contaminating DNA was checked by PCR directly on the RNAs. RNA samples were stored with RNA later, at -80°C until use. RNA concentration was determined spectrophotometrically with a Nanodrop spectrophotometer ND-1000. One microgram of total RNA was retrotranscribed into cDNA, using the ProtoScript First Strand cDNA Synthesis Kit (BioLabs, Ipswich, Massachusetts, United States) following the manufacturer’s instructions. The RT-qPCR mixture (20 μl) contained 10 μl of Light Cycler 480 SYBR Green I Master (Roche), 0.2 μM of each primer (Life Technologies-Invitrogen, Milan, Italy) and diethylpyrocarbonate-treated water (DEPC-water). One microliter cDNA (100 ng/μl) was added to each reaction mixture. The qPCR program was common for each primer set, as reported in Table 2. Calculation of relative transcript levels (RTLs) was carried out as described by Livak and Schmittgen (2001). Gene expression of planktonic cells was used as reference condition. *ALG9* and *ACT1* were used as reference genes for all RT-qPCR analyses (Teste et al., 2009). The relative stability of the reference genes was calculated using the NormFinder program (Andersen et al., 2004). All samples were processed in triplicate.

## Results

### Ability of *K. marxianus* Strains to Form Biofilm on Abiotic Surface and Adhesion Properties

Thirty-three *K. marxianus* strains were selected from a total of 83 strains based on genotypic typing and characterization from previous studies (Fasoli et al., 2016; Tittarelli et al., 2018) and they were tested for their capacity to produce biofilm on polystyrene plates and mat structures. Only 8 of them (6M2, LM142, M135, 1SC4, CBS834^T^, FM09, M83, and VG4) formed mat structures and showed a stable, dimorphic growth pattern on polystyrene in YPD and whey, with both planktonic and biofilm-forming cells. The others 25 strains were not able to adhere to polystyrene plates and to produce a mat structure, which was similar to negative control. Therefore, they were not analyzed any further.

**FIGURE 1:**
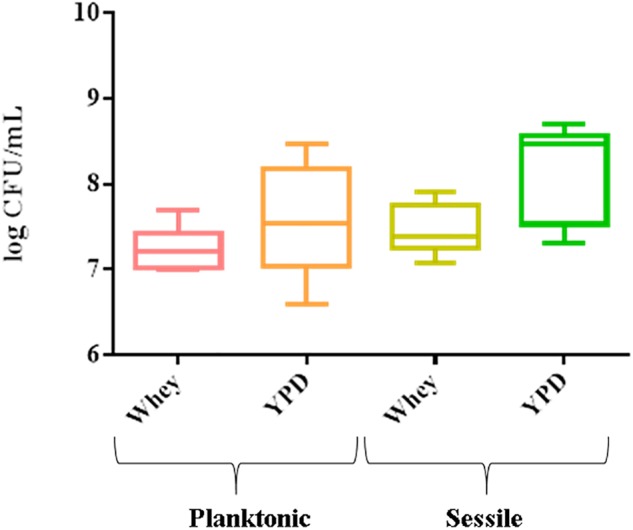
Box plot showing viable yeast cell counts of the 8 strains tested, in planktonic and sessile phase on polystyrene plates in YPD and whey. Three biological replicates were performed.

The 8 strains formed a biofilm in both substrates. The mean cell number in biofilm varied from 7.3 log CFU/mL (CBS834^T^) to 8.7 log CFU/mL (1SC4) in YPD and from 7.08 log CFU/mL (CBS834^T^) to 7.9 log CFU/mL (LM142) in whey (Figure 1). The planktonic cells ranged from 6.6 log CFU/mL (6M2) to 8.48 log CFU/mL (VG4) in YPD and from 7 log CFU/mL (1SC4 and FM09) to 7.7 log CFU/mL (VG4) in whey (Figure 1).

**FIGURE 2:**
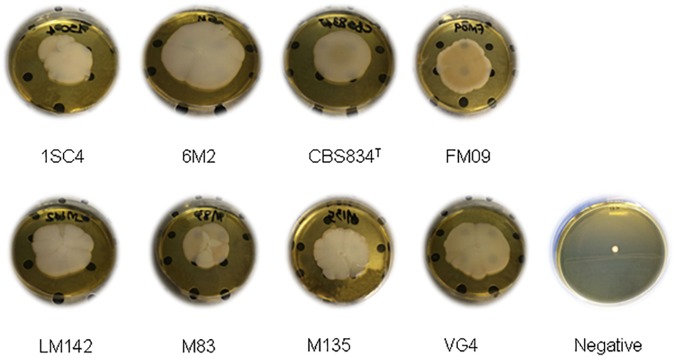
Mat formation phenotypes on soft agar.

The mat structures formed by the 8 strains were composed of a central hub with wrinkles and channels with rough edges (or rim) in YPD (Figure 2). The observed structures showed macroscopic differences in terms of diameter and edges.

**FIGURE 3:**
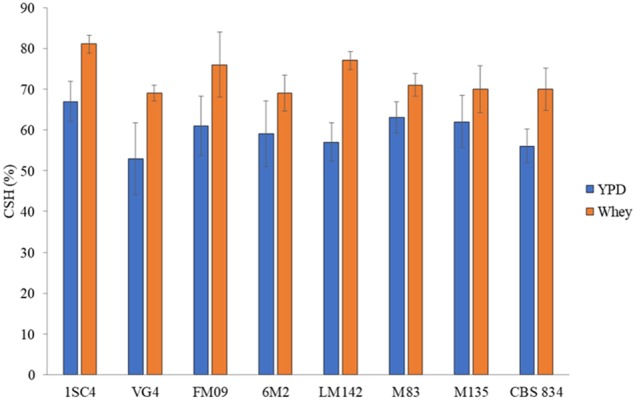
Hydrophobic nature of cells surface grown in YPD (blue) and whey (orange). Three biological replicates were performed. The error bars show the standard deviation.

The MATS test was performed to evaluate the cell surface features of selected strains grown in YPD and whey. Strains showed different level of hydrophobicity, and the highest values were detected in whey-grown cells. CSH values ranged from 55% (VG4) to 66% (1SC4) in YPD-grown cells and from 69% (VG4 and CBS834^T^) to 81% (1SC4) in whey-grown cells (Figure 3).

### Phylogenetic Analysis

**FIGURE 4:**
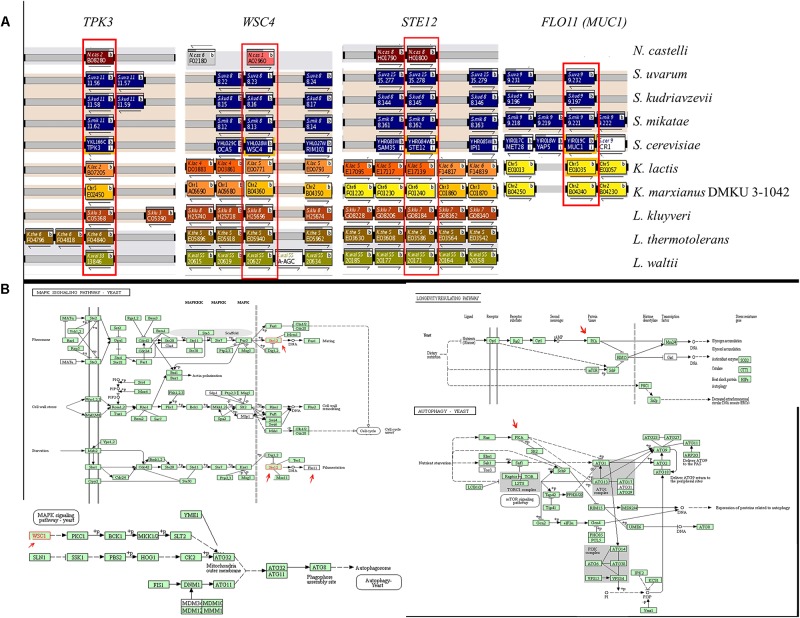
**(A)** Phylogenetic analysis of *STE12*, *FLO11*, *TPK3* and *WSC4* orthologous genes in the order Saccharomycetales. The distribution of orthologous genes was assessed using the Yeast Gene Order Browser (YGOB), which shows syntenic relationships between genomes. Genes are shown as shaded blocks with blocks of the same color, indicating that these genes are on the same chromosome. Orthologous genes are displayed in the vertical column, with gaps inserted to accommodate genes gained or lost. The *STE12*, *FLO11*, *TPK3* and *WSC4* genes are boxed and labeled. **(B)** Annotated KEGG pathway image maps. The selected genes are highlighted with a red arrow.

The genes involved in adhesion properties in some yeasts belonging to the order Saccharomycetales were studied using the YGOB, SGD or BLAST on NCBI. Orthologous of *S. cerevisiae FLO11* (also known as *MUC1*), *STE12*, *WSC4* and *TPK3* were found in most species (Figure 4A). For each selected gene, an annotated KEGG pathway image map is presented, wherein the genes are highlighted with a red arrow (Figure 4B). Multiple sequence alignment and phylogenetic analysis were carried out to compare the nucleotide sequences of the genes (Supplementary Figure S1) and the corresponding amino acidic sequences (Supplementary Figure S2). The trees showed a very strong separation of the genes. Branching of the genes clustered with generally expected patterns, but the grade of difference between the groups was high, with long branch in most clades. Thus, *K. marxianus* and *K. lactis* sequences formed a close group, different from other clades, indicating a divergent nucleotide sequence. The tree based on the amino acid sequences (Supplementary Figure S2) showed a similar pattern to the tree based on the nucleotide sequences (Supplementary Figure S1) revealing a similar codon usage of the analyzed strains.

### RT-qPCR Analysis

**FIGURE 5:**
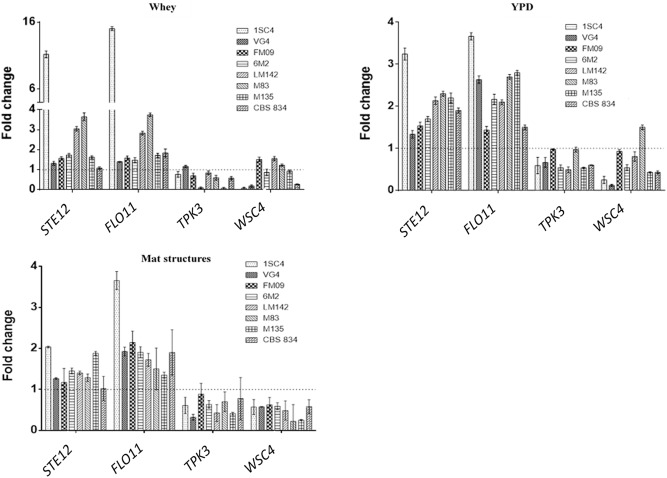
Relative transcript levels of tested genes in whey-grown cells, YPD-grown cells and mat structures. Transcript levels of each gene are expressed as the relative fold change, with planktonic cells as reference condition. Three biological replicates were performed.

Novel primer sets were developed to detect genes involved in *K. marxianus* adhesion properties. To test if the designed qPCR primers worked efficiently, calibration curves were created. Serial dilutions of cDNA were made. The calibration curves created from different dilutions of cDNA were linear and efficiencies of the qPCR reactions were calculated. Efficiency of the reaction was calculated directly from the slope of the calibration curve. All the calculated efficiencies fall within this range. Melting curves analysis revealed the specificity of designed primers: a unique peak was observed, confirming the specificity of the amplification. RT-qPCR analysis revealed interesting differences among strains. The most expressed genes were *STE12* and *FLO11* in all conditions tested. In sessile cells grown in whey these genes showed the highest fold changes in 1SC4 strain (11.21 for *STE12* and 15.05 for *FLO11*) (Figure 5). *TPK3* was upregulated only in VG4 strain (fold change = 1.16), and *WSC4* in FM09 (fold change = 1.74), in LM142 (fold change = 1.48) and M83 (fold change = 1.18). In YPD, fold changes were lower than in whey. However, also in this condition, an over expression of *STE12* and *FLO11* genes was observed in almost all strains, with values ranging from 1.3 (VG4) to 3.24 (1SC4), and from 1.43 (FM09) to 3.67 (1SC4), respectively. In this condition, *TPK3* was not upregulated, while *WSC4* was over expressed only in M83 strain (fold change = 1.51).

In mat structures the fold changes for *STE12* gene ranged from 2 (1SC4) to 1.17 (FM09), while for *FLO11* from 3.6 (1SC4) to 1.3 (M135). *WSC4* and *TPK3* were not upregulated in almost none of the tested strains in mat structures.

## Discussion

The ability of yeasts to form biofilm on food matrices, such as cheese surfaces, is essential for their growth and their involvement to the improvement of the final product quality (Mortensen et al., 2005). Many microorganisms such as *Pseudomonas aeruginosa*, *S. cerevisiae, C. albicans*, and *Candida glabrata* can form biofilms (Hawser and Douglas, 1994; Reynolds and Fink, 2001; Gaur and Klotz, 2004; Hall-Stoodley et al., 2004; Otoo et al., 2008; Bojsen et al., 2012; Granger, 2018). Genetic and phenotypic diversity, reported for cells living in biofilms, permits to have more resistance than cells in free-living planktonic form (Andersen et al., 2014). Membrane and cell wall properties seem to be modulated by environmental and nutritional factors, that may affect CSH. There are also evidences that the presence of particular proteins, such as p37, can be correlated to the ability of the *K. marxianus* cells to flocculate with high temperatures as a mechanism of response to thermic stress (Moreira et al., 1998).

Therefore, in this study 33 *K. marxianus* strains, were tested for their adhesion properties in terms of biofilm and mats formation and CSH. Pasteurized whey was considered to verify these processes in a real system, and not only in a synthetic media. It was not possible to find an association between this parameter and yeast adhesion on abiotic surfaces in agreement with other authors (Raut et al., 2010; Ichikawa et al., 2017; Perpetuini et al., 2018a), suggesting that hydrophobicity can’t be used as predictor of biofilm formation. The capacity to form mat structure and biofilm suggest the ability to adhere to industrial surfaces, which may include the type of equipment and the nature of surfaces. Moreover, it may be useful to form associations with other microorganisms. Like *S. cerevisiae* wild type strains isolated from must and grapes (Sidari et al., 2014) the *K. marxianus* strains analyzed in this work, were able to form diverse mat structures in terms of size and architecture (Figure 2). The genome differences may explain these variances as suggested by Borneman et al. (2008, 2011, 2016) for wine strains.

The molecular processes on the base of biofilm development and biofilm cell diversification in bacteria has been studied extensively, however, less is known in eukaryotic cells such as yeasts (Andersen et al., 2014). To better visualize the molecular processes, the pathways that involve the genes analyzed in this study are shown in Figure 4B.

To study a possible correlation between genotype and phenotype, we studied the expression of four genes involved in biofilm formation and stress response in *S. cerevisiae*. The overexpression of *FLO11* gene agrees with previous observations on *S. cerevisiae* (Figure 5). The *S. cerevisiae* strain S1278b can form biofilm in liquid and solid surfaces thanks to the expression of Flo11p. Other than biofilm formation, this protein is also responsible for other morphotypes, such as haploid-invasive growth and diploid-pseudohyphal growth (Reynolds and Fink, 2001). Similarly, Ste12p is required for the pseudohyphal response in *S. cerevisiae* (Gustin et al., 1998).

Obtained data revealed a possible marginal role of *TPK3* and *WSC4* on biofilm formation. Previous studies revealed that Tpk3p has two effects on biofilm formation: repression of *FLO11* transcription in biofilms and consequently loss of biofilm (Andersen et al., 2014). In *K. marxianus* under our conditions *TPK3* was not upregulated, with a subsequently expression of *FLO11* in sessile cells, in agreement with the Tpk3p role.

We analyzed the expression of the orthologous of YHL028W gene, since it shows similarity to *WSC4*, a gene involved in mat structure formation in *S. cerevisiae* (Sarode et al., 2013). However, under our conditions it showed low fold changes compared to other tested genes, suggesting the involvement of other metabolic pathways in *K. marxianus*.

## Conclusion

This study highlighted the ability of *K. marxianus* dairy strains to form biofilms not only in synthetic media but also in a real substrate such as whey. This trait could be of particular interest at industrial level since yeast adhesion on cheese surfaces is essential for its establishment and growth on the food surface. Moreover, *K. marxianus* plays an important role in cheese ripening, being essential for the contribution to the final product quality, especially for what concern the production of particular aromatic compounds (Mortensen et al., 2005; Gori et al., 2011). In addition, for the first time the overexpression of *FLO11* and *STE12* in *K. marxianus* sessile cells was shown. Since the adhesion of the yeasts is the results of several components, further studies are necessary to examine in depth the role of these genes in *K. marxianus* adhesion ability and mats formation on cheese surfaces.

## Author Contributions

GS and RT designed research and revised manuscript. GP and FT performed experiments and drafted manuscript. All authors approved final version of manuscript.

## Conflict of Interest Statement

The authors declare that the research was conducted in the absence of any commercial or financial relationships that could be construed as a potential conflict of interest.

## Footnotes

1 http://ygob.ucd.ie/

2 www.yeastgenome.org

3 https://blast.ncbi.nlm.nih.gov/Blast.cgi

4 https://www.genome.jp/kegg/genes.html

5 http://www.bioinformatics.nl/cgi-bin/primer3plus/primer3plus.cgi/

6 http://www.ebi.ac.uk/blastall/nucleotide.html

## Supplementary Material

FIGURE S1 Multiple sequence alignment of nucleotide sequences of the genes studied. A phylogenetic tree was drawn with *Neighbor-Joining* method to show relationships between the orthologous genes in yeasts (light blue: *FLO11*; Purple: *TPK3*; Red: *WSC4*; Green: *STE12*). The bootstrap analysis was conducted on 1000 replicates.

FIGURE S2 Multiple sequence alignment of amino acidic sequences of the genes studied. A phylogenetic tree was drawn with *Neighbor-Joining* method to show relationships between the orthologous genes in yeasts. The bootstrap analysis was conducted on 1000 replicate.
