# Supplementary figures and images for: Cell Wall Surface Properties of Kluyveromyces marxianus Strains From Dairy-Products
Source: Front Microbiol. 2019 Jan 31;10:79. doi: 10.3389/fmicb.2019.00079 (PMC6366010; doi:10.3389/fmicb.2019.00079)

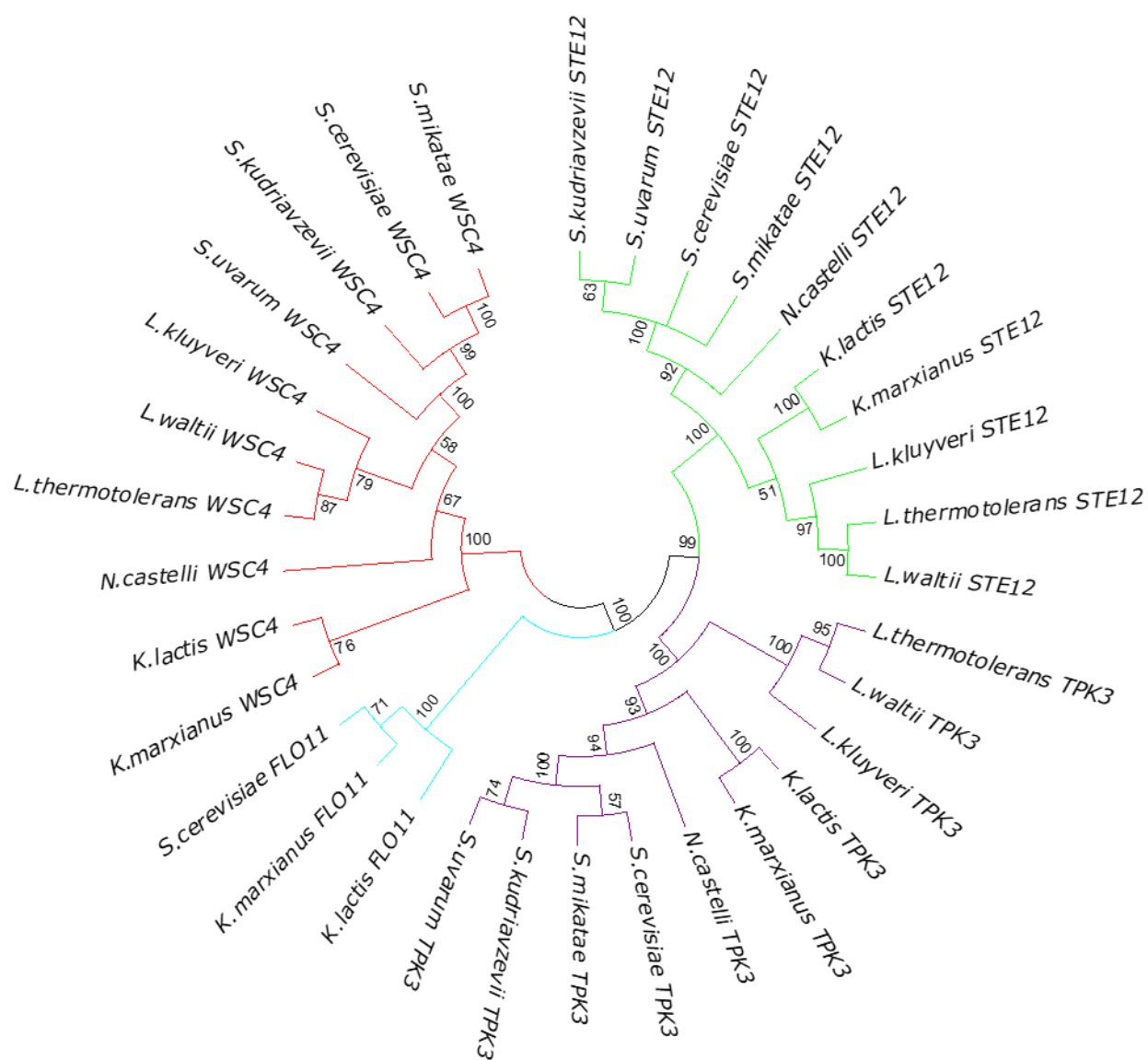

Figure S1

Supplement: FIGURE S1 — Multiple sequence alignment of nucleotide sequences of the genes studied. A phylogenetic tree was drawn with Neighbor-Joining method to show relationships between the orthologous genes in yeasts (light blue: FLO11; Purple: TPK3; Red: WSC4; Green: STE12). The bootstrap analysis was conducted on 1000 replicates. [file Data_Sheet_1.PDF]

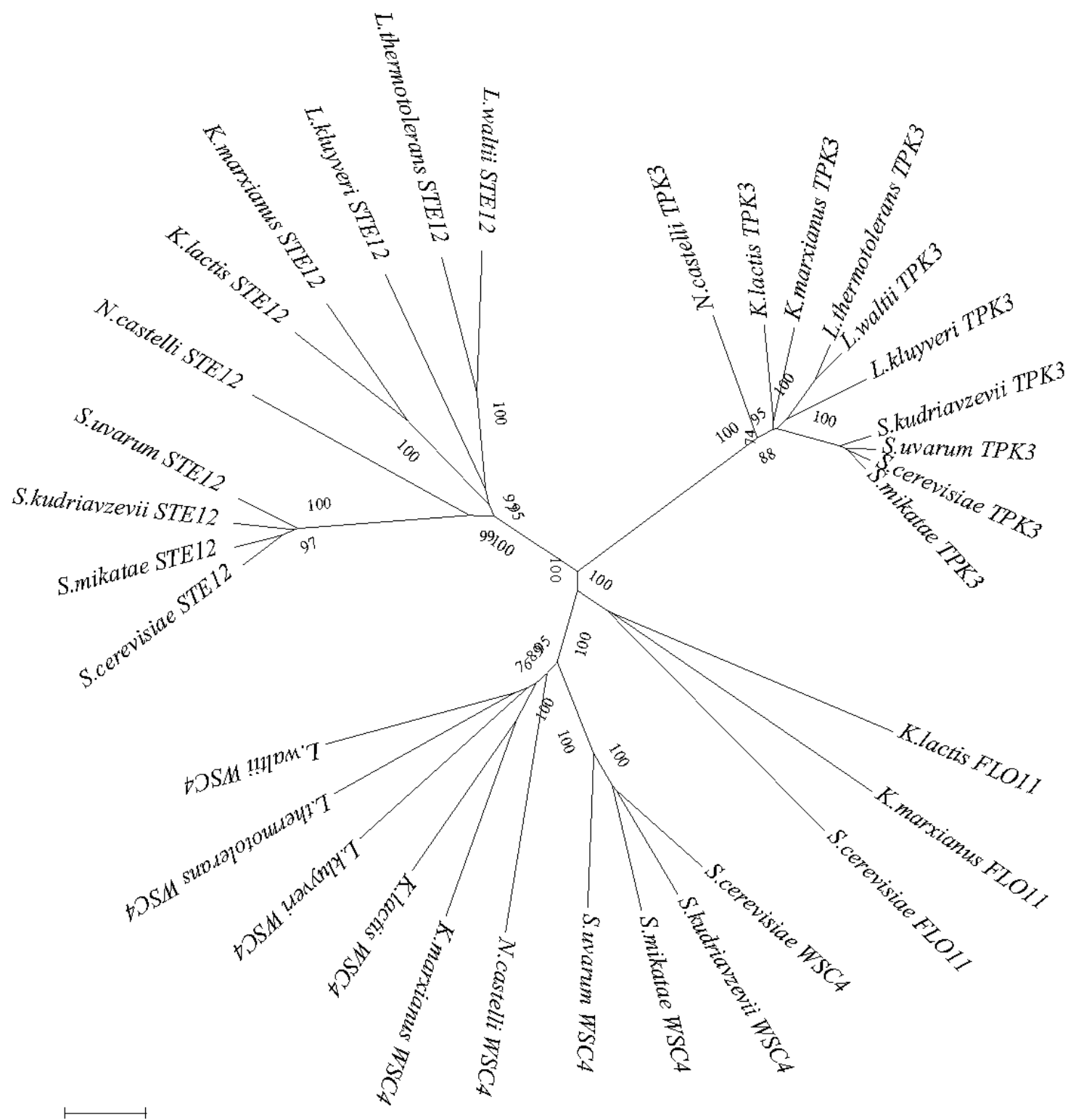

0.1

Supplement: FIGURE S2 — Multiple sequence alignment of amino acidic sequences of the genes studied. A phylogenetic tree was drawn with Neighbor-Joining method to show relationships between the orthologous genes in yeasts. The bootstrap analysis was conducted on 1000 replicate. [file Data_Sheet_2.PDF]
